# Crystal Structure of Silkworm *Bombyx mori* JHBP in Complex With 2-Methyl-2,4-Pentanediol: Plasticity of JH-Binding Pocket and Ligand-Induced Conformational Change of the Second Cavity in JHBP

**DOI:** 10.1371/journal.pone.0056261

**Published:** 2013-02-20

**Authors:** Zui Fujimoto, Rintaro Suzuki, Takahiro Shiotsuki, Wataru Tsuchiya, Akira Tase, Mitsuru Momma, Toshimasa Yamazaki

**Affiliations:** 1 Biomolecular Research Unit, National Institute of Agrobiological Sciences, Tsukuba, Ibaraki, Japan; 2 Insect Growth Regulation Research Unit, National Institute of Agrobiological Sciences, Tsukuba, Ibaraki, Japan; Centro Nacional de Biotecnologia - CSIC, Spain

## Abstract

Juvenile hormones (JHs) control a diversity of crucial life events in insects. In Lepidoptera which major agricultural pests belong to, JH signaling is critically controlled by a species-specific high-affinity, low molecular weight JH-binding protein (JHBP) in hemolymph, which transports JH from the site of its synthesis to target tissues. Hence, JHBP is expected to be an excellent target for the development of novel specific insect growth regulators (IGRs) and insecticides. A better understanding of the structural biology of JHBP should pave the way for the structure-based drug design of such compounds. Here, we report the crystal structure of the silkworm *Bombyx mori* JHBP in complex with two molecules of 2-methyl-2,4-pentanediol (MPD), one molecule (MPD1) bound in the JH-binding pocket while the other (MPD2) in a second cavity. Detailed comparison with the apo-JHBP and JHBP-JH II complex structures previously reported by us led to a number of intriguing findings. First, the JH-binding pocket changes its size in a ligand-dependent manner due to flexibility of the gate α1 helix. Second, MPD1 mimics interactions of the epoxide moiety of JH previously observed in the JHBP-JH complex, and MPD can compete with JH in binding to the JH-binding pocket. We also confirmed that methoprene, which has an MPD-like structure, inhibits the complex formation between JHBP and JH while the unepoxydated JH III (methyl farnesoate) does not. These findings may open the door to the development of novel IGRs targeted against JHBP. Third, binding of MPD to the second cavity of JHBP induces significant conformational changes accompanied with a cavity expansion. This finding, together with MPD2-JHBP interaction mechanism identified in the JHBP-MPD complex, should provide important guidance in the search for the natural ligand of the second cavity.

## Introduction

Juvenile hormones (JHs) are acyclic sesquiterpenoids which contain an α,β-unsaturated methyl ester and a terpenoid backbone with an epoxide distal to the ester. Both the ester and epoxide groups are required for hormone regulatory functions. JH regulates diverse processes including the growth, development, metamorphosis and reproduction of insects [1], [2]. The JH levels control the full cycle of insect development from the immature larval stage to the adult form. Hence, insect growth regulators (IGRs) with JH-agonistic or -antagonistic activity have become attractive candidates for insect pest control since the identification of the structure of JH in 1967 [3]. Several JH analogues (JHAs) such as methoprene [4], fenoxycarb [5] and pyriproxyfen [6] have been proven to be effective IGRs and useful as insecticides against household and agricultural pests that cause damage worth billions of pounds to global agriculture each year.

The diversity of JH-mediated physiological effects suggests that target cells may respond to the hormone directly by gene expression and/or via a second messenger [7], [8]. Since JH actions for the individual processes occur in tissue- and stage-specific manner, it is generally assumed that multiple numbers of JH receptors exist at the membranes and in cytosols and nuclei. Upon release from the *corpora allata* where JH is synthesized, the hormone is dispersed to the hemolymph to act at distant peripheral sites. In Lepidoptera almost every molecule of JH in the insect hemolymph appears in a complex with a specific 30 kDa JH-binding protein (JHBP) which serves as a carrier supplying the hormone to target cells [9]–[12]. Complex formation provides protection of the chemically labile JH against nonspecific enzymatic degradation and/or sequestration, and is crucial for effective signaling by the low amounts of the hormone. In this sense, JHBP is one of the most important proteins that regulate the JH signaling.

**Figure 1:**
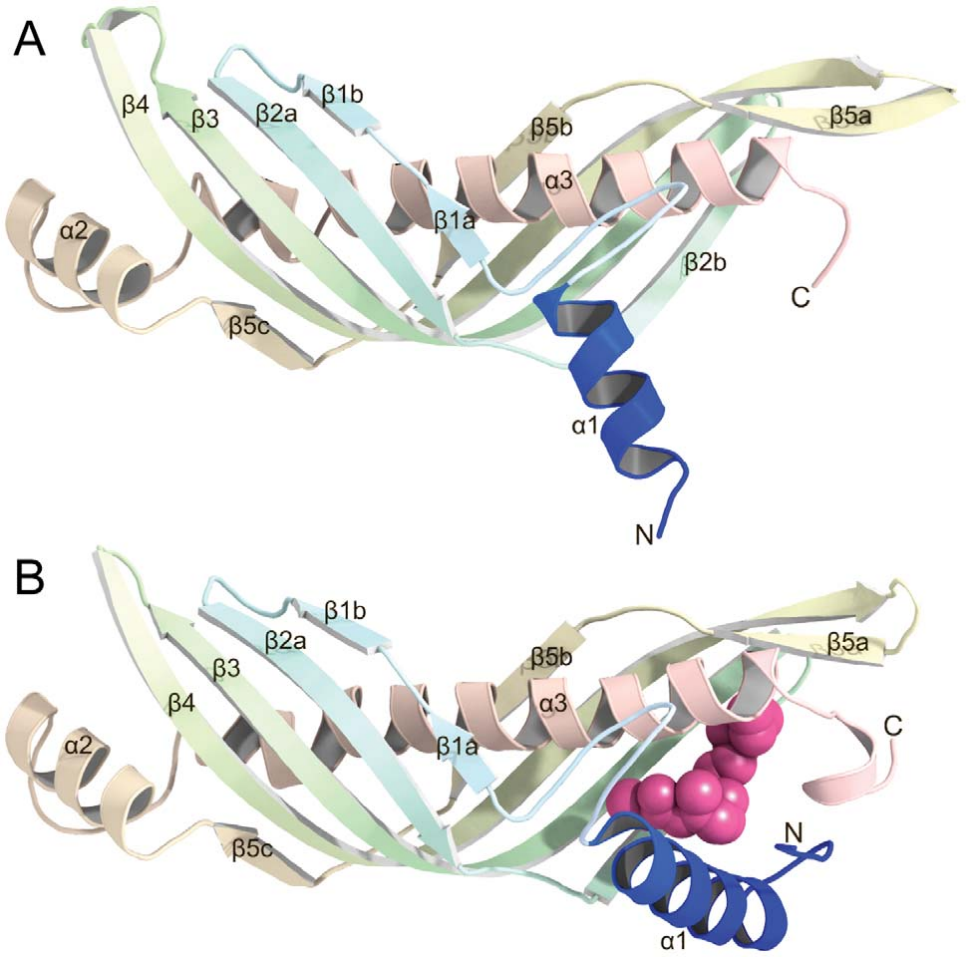
Crystal structures of *Bombyx mori* JHBP. (A) A gate-open conformation of JHBP in the apo-form. The gate helix α1 shown in blue resides in an open conformation which permits access of JH to the preserved hormone-binding pocket. (B) A fully gate-closed conformation of JHBP in complex with JH II. The bound JH II molecule is shown as a space-filling model. The gate helix α1 shown in blue covers the hormone-binding pocket to maintain the bound JH II molecule deep inside the protein.

In previous reports [13], [14], we have reported a gate-latch mechanism of JH delivery in hemolymph by JHBP based upon the crystal and solution structures of apo- and JH-bound forms of the recombinant JHBP from the silkworm, *Bombyx mori*. JHBP adopts a unique elongated β-barrel fold, consisting of a long spine helix (α3) wrapped in a highly curved β-sheet, which is suitable for a transport of the hydrophobic JH (Figure 1). Nearly the same folds are shared by several other lipid binding proteins: Takeout, a potential ubiquinone binding protein [15]; a bactericidal permeability-increasing protein [16]; and a cholesteryl ester transfer protein [17]. JH binds to a hydrophobic JH-binding pocket located at the one end of the elongated structure near the C-terminus of α3. The uptake and release of JH are regulated by the opening and closing of the α1-helix over the JH-binding pocket that functions as a gate sensing the ligand entry. In the crystal structure of the apo-JHBP (gate-open conformation), the location of the α1-helix generates a wide open conformation which permits access of JH to the hormone-binding site (Figure 1A). Binding of the JH molecule induces a dramatic change in the orientation of α1, which swings towards the pocket about 70° from the position of the corresponding helix in the apo-structure. In the resulting fully gate-closed JHBP-JH complex structure (Figure 1B), the bound JH is completely buried inside the protein and is thus protected from unfavorable nonspecific absorption and enzymatic degradation during its transport in the hemolymph. In solution, the apo-JHBP assumes multiple conformations of the gate α1-helix ranging from the fully closed to open forms while the protein core is well maintained in all of these structures. JH binding silences conformational dynamics of the α1 gate and leads to the formation of the unique conformation seen in the JHBP-JH complex [13]. Our dynamic structural model visualizes the JH-induced conformational change of JHBP previously reported by a range of different techniques such as proteolysis and ultracentrifugation [18], UV-difference and CD spectroscopy [12], [19], and electrochemical impedance spectroscopy [20].

Structural studies revealed that JHBP has an additional hydrophobic cavity (second cavity) on the side opposite from the JH-binding pocket [13], [21]. In both the apo- and JH-bound structures of the recombinant *B. mori* JHBP, this second cavity remains empty [13]. In contrast, ligand binding to the second cavity is suggested by the crystal structure of the native JHBP from the wax moth *Galleria mellonella* in which the second cavity accommodates an undefined small molecule while the JH-binding pocket remains empty [21]. Although the nature of the second ligand is not known, it was speculated that the second ligand could be bound to the cell membrane to allow for hormone delivery site recognition by JHBP [21]. Further studies are necessary to validate the accurate functional roles of the second ligand-binding site.

Lepidopteran insects are major agricultural pests. For example, the tobacco hornworm *Manduca sexta* and the tobacco budworm *Heliothis virescens* are the most destructive pests of tobacco [22], while the wax moth *G. mellonella* causes serious damage to beehives [23]. Since the hemolymph JHBP mediates the first step in the JH signal transduction cascade, and is highly specific to Lepidopteran insects, JHBP is expected to be an excellent target for the design of novel specific IGRs and insecticides. The first three-dimensional structures of the JHBP-JH II complex in the crystalline state and the JHBP-JH III complex in solution, together with subsequent biochemical assays, enabled us to elucidate the molecular mechanism of JH recognition by JHBP that clearly explains the ligand specificity and enantioselectivity [13], [24]–[28]. The structural information derived from the JHBP-JH complexes opens the way to the structure-based design of IGRs which inhibit complex formation between JH and JHBP, and thus disrupt the JH signaling in Lepidoptera. The development of such IGRs should be further accelerated by enhancement of our understanding of interactions of JHBP with any ligands structurally related and unrelated to the JH molecule.

Here we report the crystal structure of the recombinant *B. mori* JHBP in complex with 2-methyl-2,4-pentanediol (MPD). This is the first structure of JHBP in complex with a ligand which is structurally unrelated to JH. In this structure, we found two bound MPD molecules, one (MPD1) in the JH-binding pocket and the other (MPD2) in a second cavity. MPD1 is anchored in the same hydrophobic cage as the epoxide of the JHBP-bound JH. Furthermore, interactions of MPD1 with JHBP are nearly the same as those observed for the epoxide moiety of JH in the JHBP-JH complex. We confirm that MPD and methoprene containing a MPD-like structural element, but not the unepoxydated JH III (methyl farnesoate), inhibit the binding of JH to JHBP using the ligand competitive binding assay. These findings strongly demonstrate that the epoxy group and its mimic structures are critically important for the ligand binding to the JH-binding pocket of JHBP, and should provide useful information for IGR design targeted for JHBP. We also found that MPD2 binding induces a significant conformational change in the second cavity. The MPD2-JHBP interactions reported here should provide important guidance in the search for the natural ligand of the second cavity.

## Results and Discussion

### Overall Structure of the JHBP-MPD Complex

**Figure 2:**
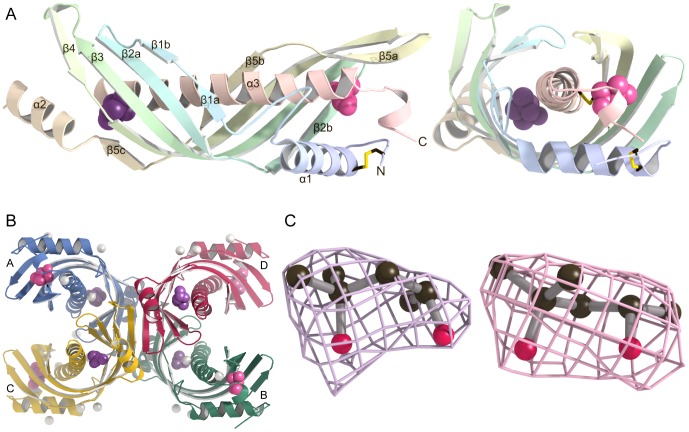
Crystal structure of JHBP-MPD complex. (A) Two views of a ribbon diagram illustrating the protein folding. The bound MPD molecules, MPD1 (pink) in the JH-binding pocket and MPD2 (purple) in the second cavity, are shown as space-filling models and disulfide bonds as stick models. (B) A ribbon diagram of the four JHBP-MPD complexes in the asymmetric unit. Zinc ions are indicated by white spheres, MPD by space-filling models. (C) The |Fo| – |Fc| omit electron density maps of MPD1 (right) and MPD2 (left) contoured at 1σ. Carbon and oxygen atoms are shown in black and red, respectively.

The crystal structure of *B. mori* JHBP in complex with MPD was solved at a resolution of 2.6 Å by a single-wavelength anomalous dispersion (SAD) method using selenomethionine (SeMet)-substituted crystals (Figure 2 and Table 1). As shown in Figure 2A, the MPD-bound JHBP adopts a gate-closed conformation which is similar to the structure of the JH-bound JHBP [13]. The refined model contains four JHBP monomers (A, B, C and D) in the crystal asymmetric unit, which are related by 222 point group symmetry (Figure 2B). No stable dimerization could be seen in any two pairs of the four NCS-related molecules. Consistent with this, gel filtration and ultracentrifugation experiments as well as NMR spectroscopy have shown unambiguously that JHBP is monomeric in solution [13], [14], [18], [29], [30].

**Table 1:** Summary of data collection and refinement statistics of the crystal structures of the JHBP-MPD complex.

	MPD
**Data collection**	
Space group	*P*2_1_2_1_2_1_
Unit cell parameters	
* a* (Å)	54.9
* b* (Å)	114.7
* c* (Å)	192.9
Beam Line	PF 17A
Wavelength (Å)	0.97000
Resolution (Å)	50–2.60(2.69–2.60)
Total reflections	473812
Unique reflections	38636 (3701)
*R*-merge	0.099(0.489)
Completeness (%)	99.7(97.5)
Average *I/σ(I)*	22.2(3.1)
Average redundancy	12.3(9.2)
**Structure refinement**	
Resolution (Å)	49.5–2.59(2.66–2.59)
*R*-factor	0.222(0.310)
*R*_free_-factor^a^	0.290(0.382)
RMSD from ideal	
Bond lengths (Å)	0.016
Bond angles (°)	1.75
Ramachandran plot (%)	
Favoured regions	92.2
Allowed regions	5.6
Outlier regions	2.2

a *R*_free_-factors were calculated using 5% of the unique reflections.

We could trace the electron density of residues 7–174 and 183–222 for molecule A, of 7–175 and 180–222 for molecule B, of 5–175 and 183–223 for molecule C, and of 7–174 and 182–222 for molecule D. The four molecules overlay well with one another, giving root mean square difference (RMSD) values of less than 0.70 Å for C^α^ atoms between any two pairs of them. For all the four JHBP molecules, we revealed two bound MPD molecules, one (MPD1) in the JH-binding pocket and the other (MPD2) in the second cavity at each end of the elongated structure (Figures 2A and C). These MPD molecules were most likely incorporated from the precipitant solution during crystallization of the apoprotein.

### Structural Plasticity of the JH Binding Pocket in JHBP

**Figure 3:**
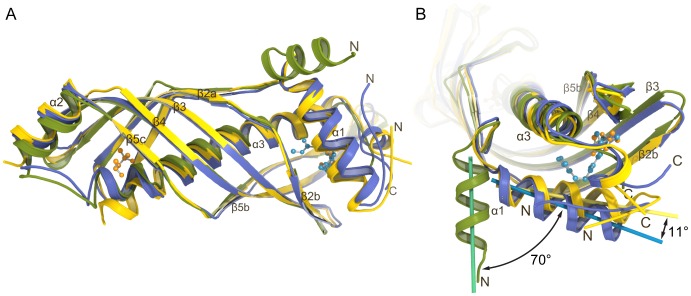
Comparison of the MPD-bound JHBP structure with the apo- and JH II-bound JHBP structures. (A) A side-view of the overlay of the crystal structures of the MPD-bound (yellow), JH II-bound (blue) and apo-JHBP (green), showing overall superposition. Significant deviations are observed at both ends of the elongated structure. The bound MPD and JH II molecules are shown as ball-and-stick models. (B) A top-view of the same overlay illustrates the difference around the JH-binding pocket. Axes of α1 helices are also shown. The orientation of the α1 helix on the JH-binding pocket, that functions as a gate sensing the ligand binding, is significantly different between the three states.

Comparison of the MPD-bound and JH II-bound JHBP structures reveals that the core of the protein assumes nearly the same structure with RMSD values ranging from 0.69 to 1.05 Å for C^α^ atoms of residues 32–172 and 190–210 depending on the pair of chains superposed (Figure 3A). However, pronounced displacements are observed at both ends of the elongated structure. The largest displacement involves the gate α1 helix which covers the JH-binding pocket (Figure 3B). The structural rearrangement of α1 is correlated with the difference in the molecular size between JH and MPD. Comparing with the JH II-bound structure, the α1 helix of the MPD-bound structure is folded back into the hormone binding pocket about 11° and generates contacts of the Leu17 and Thr21 side chains in helix α1 with MPD, which is smaller in size than JH. Concomitantly, the N-terminal arm and the C-terminal tail that serve as a latch for the JH-binding pocket are pushed out toward solvent. When compared with the unliganded apo-JHBP structure [13], the movement of the α1 helix is 81° (Figure 3B).

**Figure 4:**
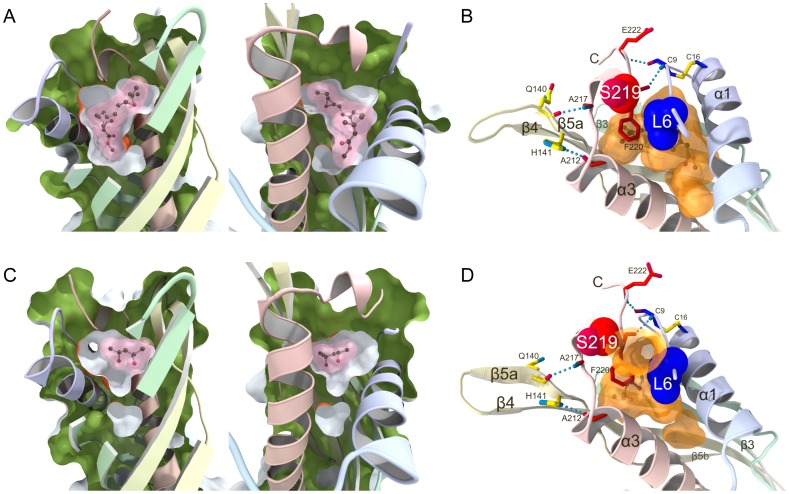
Structural plasticity of the JH-binding pocket in JHBP. (A) Views of the JH-binding pocket of the crystal structure of JHBP in complex with JH II. The figure is drawn for the JH-complex A, one of the two complexes in the crystal asymmetric unit. Surface representations of the complex structure were split vertically through the JH-binding pocket perpendicular to the page. The halves produced from the split were rotated in opposite directions to create the views shown. The interior of the protein and the exterior of the pocket are colored green and orange, respectively. The bound JH II molecule is shown as a ball-and-stick model with its molecular surface (pink). (B) Interactions between the latch-forming N-terminal arm and the C-terminal tail in the JH-bound JHBP observed for JH-complex A. The N-terminal arm and C-terminal tail are further linked to the gate α1 helix by the Cys9-Cys16 disulfide bond and the protein core by hydrogen bonds (light-blue dotted lines), respectively. Key residues for interactions are shown as stick models with hydrogen bonds (light-blue dotted lines). Leu6 in the N-terminal arm and Ser219 in the C-terminal tail are shown as space-filling models. The pocket is shown as a transparent orange surface with the internal JH II molecule as a ball-and-stick model. (C) Views of the JH-binding pocket of the crystal structure of JHBP in complex with MPD. The figure is drawn for MPD-complex C, one of the four complexes in the crystal asymmetric unit. Surface representations were created as for (A). The bound MPD molecule is shown as a ball-and-stick model with its molecular surface (pink). (D) Interactions between the latch-forming N-terminal arm and the C-terminal tail in the MPD-bound JHBP observed for MPD-complex C. As in the JH-complex, the N-terminal arm and C-terminal tail are further linked to the gate α1 helix by the Cys9-Cys16 disulfide bond and the protein core by hydrogen bonds (light-blue dotted lines), respectively. Key residues for interactions are shown as stick models with hydrogen bonds (light-blue dotted lines). Leu6 in the N-terminal arm and Ser219 in the C-terminal tail are shown as space-filling models. The pockets are shown as transparent orange surfaces with the internal MPD molecule as a ball-and-stick model.

Reflecting differences in the orientation of the gate α1 helix, the sizes of the JH-binding pockets are different between the MPD-bound and JH II-bound forms. According to the Swiss-PbdViewer version 4.1 (http://www.expasy.org/spdbv) [31], the JH-binding pocket of the JH II-bound form is a huge continuous cavity. The volume was calculated to be 874 and 903 Å^3^ for the two JH-complexes A and B in the crystal asymmetric unit, respectively. The cavity is completely closed, and matches almost accurately the van der Waals surface of the bound JH II molecule as shown in Figure 4A (drawn for JH-complex A). The cavity is sealed by two hydrogen bonds, Cys9 NH-Phe220 CO and Glu222 NH-Cys9 CO, between the N-terminal arm and the C-terminal tail (Figure 4B). In contrast, the JH-binding pocket in the MPD-bound form is separated into two smaller cavities, a large one with a volume of 507 Å^3^ at the upper part which accommodates the MPD1 molecule and a tiny one with a volume of 56 Å^3^ at the bottom wall as shown in Figure 4C (drawn for MPD-complex C). The latter tiny cavity corresponds to the open space below the methyl ester of the bound JH in the JHBP-JH complex. The former large cavity is slightly expanded upward compared with that of the JH-bound form, and has an open hole due to the structural rearrangements of the α1 helix, N-terminal arm, and C-terminal tail as mentioned above. It is noteworthy, however, that the Cys9 NH-Phe220 CO and Glu222 NH-Cys9 CO hydrogen bonds are still maintained in the MPD-bound structure (Figure 4D). These observations suggest a high adaptability of the JH binding pocket which could change its size and shape in a ligand-dependent manner due to flexibility of the gate α1 helix.

### MPD Mimics Epoxide of JH

**Figure 5:**
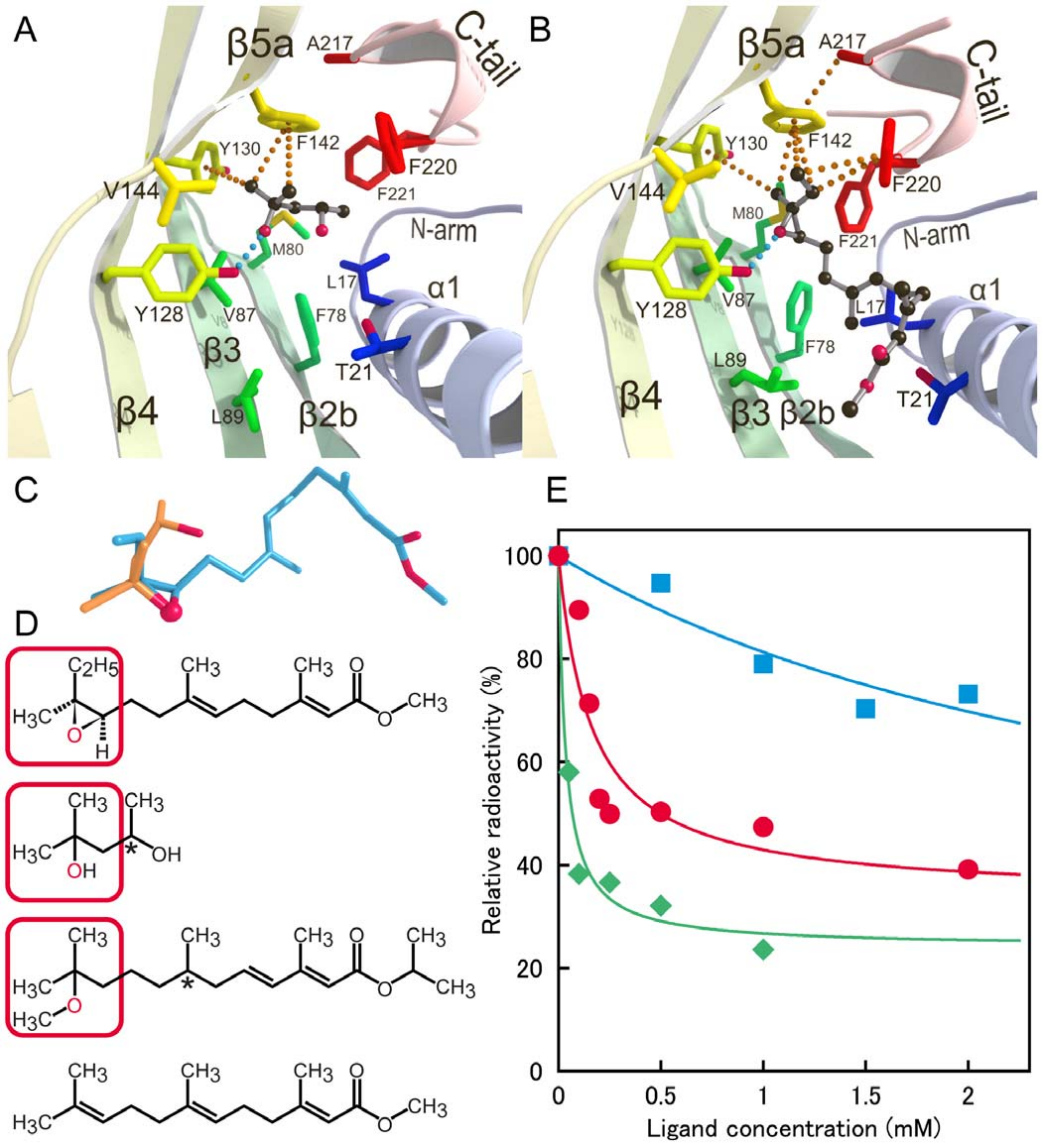
Molecular basis for MPD binding to the JH-binding pocket of JHBP. (A) Interactions between JHBP and MPD1 bound in the JH-binding pocket observed for MPD-complex C. Residues involved in the recognition are shown as stick models and MPD1 as a ball-and-stick model. Hydrogen bond and CH-π stacking interactions used for recognition of MPD1 are indicated by light blue and orange dotted lines, respectively. (B) For comparison with (A), interactions of JHBP with the epoxy moiety of JH II observed for JH-complex A are displayed where JH II is shown as a ball-and-stick model. (C) Overlay of the JHBP-bound MPD1 (orange) and JH II (light blue) molecules. The red ball represents the oxygen atom which forms an intermolecular hydrogen bond with Tyr128 O^η^H of JHBP. (D) Chemical structures of JH II, MPD, methoprene, and methyl farnesoate (MF), the unepoxidated form of JH III, are shown from the top. The asterisk denotes a chiral carbon atom. (E) Competitive binding assay for MPD, methoprene and MF. The inhibition of JH binding to JHBP by the tested ligand was followed by monitoring the reduction of the radioactivity of the JHBP-bound ^3^H-labeled JH III. The relative radioactivity is plotted as a function of the ligand concentration: MPD (filled circles in red), methoprene (filled diamonds in green), and MF (filled squares in light-blue). The values represent the average for two to six experiments.

The MPD molecule (MPD1) bound to the JH-binding pocket is confined in a hydrophobic cage formed by Phe78 and Met80 in β2b, Val87 and Leu89 in β3, Tyr128 and Tyr130 in β4, Phe142 and Val144 in β5a, and Phe220 and Phe221 in the C-terminal tail as shown in Figure 5A (drawn for MPD-complex C). The same cage accommodates the epoxide group of JH in the JHBP-JH II complex [13] as shown in Figure 5B (drawn for JH-complex A). In fact, MPD1 overlaps the epoxide group of JH II in the complex with JHBP as shown in Figure 5C. Furthermore, the recognition mode of MPD1 is essentially the same as that of the epoxide group of JH (Figures 5A and B). MPD1 is anchored in the cage by a direct hydrogen bond of the O^2^H group of MPD with the hydroxyl group of Tyr128 which forms a direct hydrogen bond with the epoxy oxygen of JH in the JHBP-JH II complex. In addition, MPD1 is further stabilized by CH-π stacking interactions between two methyl groups attached to C^2^ of MPD and the aromatic side chains of Tyr130 and Phe142. These two aromatic residues make similar CH-π stacking interactions with the methyl and ethyl groups attached to the epoxide of JH II in the JHBP-JH II complex.

Similarity in the binding mode between MPD1 and the epoxide moiety of JH raises the question if MPD competes with the binding of JH to JHBP. To answer this question, we performed a ligand competition binding assay by using a described method [32]. Binding of ^3^H-JH III to JHBP was measured in the absence and presence of MPD. For comparison, we also tested methyl farnesoate (MF) which is the unepoxidated form of JH III and JH-agonist methoprene which can be used as an insecticide. Figure 5D shows chemical structures of the tested ligands as well as JH II. Since the local structure of methoprene around the methoxy group resembles the MPD structure, the methoxy oxygen is expected to act as the hydrogen-bond acceptor from the Tyr128 of JHBP. It has been reported by electrochemical impedance spectroscopy that methoprene binds to the recombinant *G. mellonella* JHBP with the dissociation constant (*K*_d_) of 33.3 nM which is comparable to the *K*_d_ values for JH I (28.6 nM) and JH III (43.5 nM) [20].

As shown in Figure 5E, MPD inhibits the binding of ^3^H-labeled JH III to JHBP in a dose-dependent manner. The IC_50_ value was determined to be 160 µM by least-square-fitting to the experimental data using Eq. 1 described in Materials & Methods. Methoprene displayed 5-fold stronger inhibitory activity (IC_50_ = 35 µM) than MPD likely due to additional interactions with JHBP. The curve-fitting also provided the maximum reduction in the radioactivity, ΔF_max_ in Eq. 1, 66% for MPD and 76% for methoprene, which are comparable to the value for JH III (68%) estimated by use of the same binding assay [32]. In contrast with MPD and methoprene, MF inhibited only 30% of the ^3^H-JH III binding to JHBP even at the concentration of 2 mM (Figure 5E). The weak inhibitory activity of MF could be attributed to the lack of the epoxy group because the structure of the remaining part is the same as JH. Hence the results of our binding assay strongly demonstrate that the epoxy group and its mimic structures, particularly the existence of the hydrogen-bond acceptor from the Tyr128 O^η^H, are critically important for the ligand binding to the JH-binding pocket of JHBP.

The dissociation constant (*K*_d_) for the tested ligand can be theoretically calculated from the IC_50_ value if the amount of the unliganded JHBP is negligible. For the ligand competition assay, we employed the condition that JHBP exists much more than JH as in the insect hemolymph (800 nM JHBP and 10 nM ^3^H-JH III in our case). It is, therefore, difficult to estimate the precise *K*_d_ value from the IC_50_ value obtained from our assay. Applying the reported *K*_d_ values of JH III (43.5 nM) and methoprene (33.3 nM) [20] to the equation of *K*_d_ = IC_50_/(1+ [JH]/*K*_d,JH_), the IC_50_ of methoprene is theoretically calculated to be 41 nM which is three-order of magnitude smaller than the IC_50_ value (35 µM) obtained by our assay. Assuming that this three-order difference between the theoretical and our observed IC_50_ values is held in the case of MPD, the *K*_d_ value is estimated to be 152 nM. This value might be the maximum because MPD can also bind to the second cavity of JHBP.

### Ligand-induced Conformational Change of the Second Cavity in JHBP

JHBP has a second hydrophobic cavity located on the side opposite from the JH-binding pocket. The second cavity is formed by the α2-helix, the N-terminal portion of the α3-helix, and the inner side of the highly curved β-sheet. The entrance of the cavity is formed by the β5c strand, the α2-helix, the N-terminal portion of the α3-helix, and presumably the α2-α3 loop, which is not observable for the MPD-bound JHBP because of the flexibility of the loop or conformational heterogeneity. The end of the β5c strand and the whole α2-helix forms one side-wall while the N-terminal portion of the α3-helix forms the other side-wall. Seven residues, Val50, Phe62, Tyr116, Ile159, Arg189, Ala192 and Ile193, form the inside-wall which closes the cavity at the middle of the protein structure. In contrast, the outside-part at the end of the second cavity remains open.

**Figure 6:**
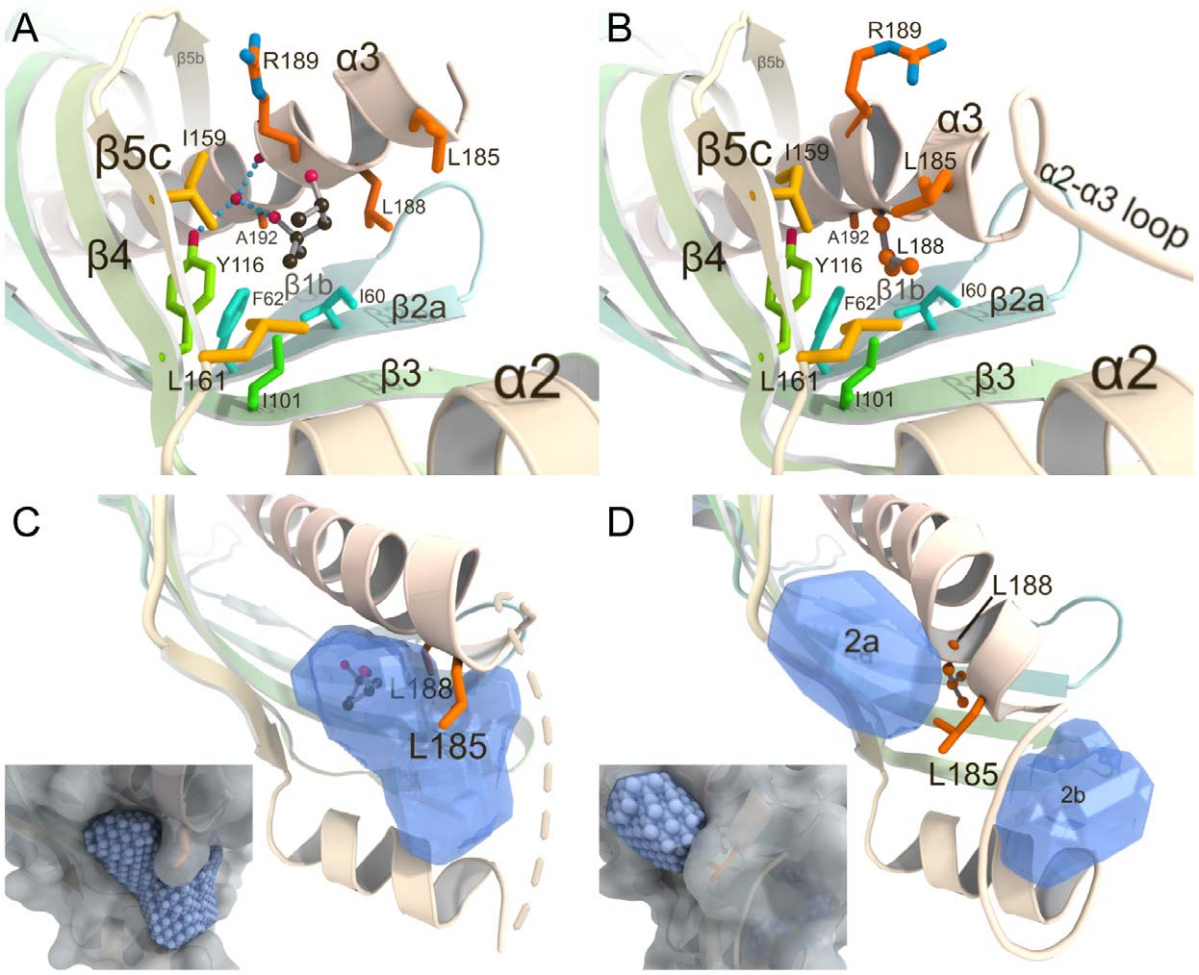
MPD-induced conformational change in the second ligand-binding cavity of JHBP. (A) A close-up view of the MPD-bound second cavity in the JHBP-MPD complex. The figure is drawn for MPD-complex A, one of the four complexes in the crystal asymmetric unit. Residues involved in the recognition of MPD are shown as stick models and the bound ligand (MPD2) as a ball-and-stick model. A water molecule and hydrogen bonds are indicated by red sphere and light-blue dotted lines, respectively. (B) A close-up view of the unliganded second cavity in the JHBP-JH II complex shows that the side chain of Leu188 shown as a ball-and-stick model occupies the same space as MPD2 in the MPD-bound structure, caused by a kinked conformation of the α3 helix. The figure is drawn for JH-complex A, one of the two complexes in the crystal asymmetric unit. (C and D) The shapes of the MPD-bound second cavity and the unliganded second cavity calculated by the program GHECOM are shown as blue transparent shells, respectively. Original grid data of GHECOM represented by spheres are shown with white molecular surfaces of the proteins in insets.

Comparison of the MPD-bound JHBP structure with the crystal structures of the apo- and JH II-bound JHBP [13] suggests a ligand-induced structural rearrangement of the second cavity. This cavity accommodates one MPD molecule (MPD2) in the MPD-bound form (Figure 6A) but remains empty in the apo- and JH-bound forms [13] (Figure 6B). Figures 6A and 6B are drawn for the MPD-complex A and the JH-complex A, respectively. There is essentially no difference in the structure of the unliganded second cavity between the apo- and JH II-bound forms. MPD2 is partially solvent-exposed, and is held in the cavity by a water-mediated hydrogen-bond network with Tyr116 O^η^H and Arg189 CO, and hydrophobic interactions with Ile60 and Phe62 in β2a, Ile101 in β3, Tyr116 in β4, Ile159 and Leu161 in β5c, and Leu185, Leu189, Arg189 and Ala192 at the N-terminus of helix α3 (Figure 6A). The α3-helix has no kink in its conformation in the MPD-bound second cavity (Figure 6A), but is kinked at position 190 near the N-terminus in the unliganded second cavity (Figure 6B), where the side chain of Leu188 occupies the same space as MPD2 in the MPD-bound structure. In addition, a loop connecting helices α2 and α3 is visible in the apo- and JH II-bound forms. As a result, the unliganded second cavity is shallow when compared to the liganded one.

Ligand binding to the second cavity is also suggested by the crystal structure of the native *G. mellonella* JHBP [21]. In this structure, the second cavity accommodates an undefined small molecule for which a 7 Å long patch of electron density starts from a point about 3 Å from the functional groups of Lys51, Tyr62 and Thr193 toward the main entrance. The equivalent residues in *B. mori* JHBP are hydrophobic: Val50, Phe62 and Ala192. It is worth mentioning that the starting point of this undefined molecule seems to match the location of MPD2 in our JHBP-MPD complex structure. Furthermore, in the *G. mellonella* JHBP structure long spine α4 helix, which corresponds to the α3 helix in the *B. mori* JHBP, has no kink in its conformation as the MPD-bound structure of *B. mori* JHBP reported here. Unlike the liganded and unliganded *B. mori* JHBP, the α2 and α4 helices of *G. mellonella* JHBP are connected by a clearly observable extra helix α3.

The structural change of the α3 helix of the *B. mori* JHBP from the kinked conformation to the straight one after ligand binding to the second cavity results in a cavity expansion. We used the GHECOM program (http://strcomp.protein.osaka-u.ac.jp/ghecom/) [33] to evaluate the volume of the second cavity instead of the Swiss-PbdViewer program [31] used for the JH-binding pocket because the former program provides more reasonable results for shallow pockets like the second cavity. The GHECOM analysis revealed that the volume of the MPD-bound second cavity of the *B. mori* JHBP is in a range of 741–929 Å^3^ (Figure 6C) while the unliganded second cavity of the same protein is separated into two smaller cavities with volumes of 86 (site 2a) and 233 (site 2b) Å^3^ by the Leu185 side chain in the kinked α3 helix (Figure 6D). It has been reported that the volume of the liganded second cavity of the *G. mellonella* JHBP is estimated to be 668 Å^3^ by the CASTp analysis [21], [34]. Our GHECOM [33] analysis of the same structure provided the volume of 706 Å^3^ for the second cavity of the *G. mellonella* JHBP. Hence, we suggest that binding of a small molecule to the second cavity of JHBP straightens the long spine helix, α3 in *B. mori* JHBP or α4 in *G. mellonella* JHBP, and creates a proper space for the ligand. This finding, together with the MPD2-JHBP interaction mechanism, should assist in identification of the natural ligand(s) for the second cavity of JHBP.

### Conclusion

In this paper, we present the crystal structure of the recombinant *B. mori* JHBP in complex with two molecules of MPD (MPD1 and MPD2), where MPD1 is bound in the JH-binding pocket and MPD2 in a second hydrophobic cavity. They are at different ends of the elongated protein structure. This is the first structure of JHBP in complex with a ligand which is structurally unrelated to JH. Detailed comparison with the apo-JHBP and JHBP-JH II complex structures previously reported by us [13] led to a number of intriguing findings. First, the JH-binding pocket changes its size and shape in a ligand-dependent manner due to flexibility of the gate α1 helix. Second, MPD1 mimics interactions of the epoxide moiety of JH previously observed in the JHBP-JH complex, and MPD can compete with JH in binding to the JH-binding pocket. We also confirmed that methoprene, with an MPD-like structural element, inhibits the complex formation between JH and JHBP. The existence of the hydrogen-bond acceptor from Tyr128 is critical for ligand binding to the JH-binding pocket, because methyl farnesoate, which is structurally similar to JH but lacks such acceptor, showed significantly weaker binding to JHBP. These findings could open the door to the structure-based design of novel Lepidoptera-specific IGRs which inhibit complex formation between JH and JHBP, and thus disrupt JH signaling. Third, binding of MPD to the second cavity of JHBP induces significant conformational changes accompanied with cavity expansion. This finding, together with the MPD2-JHBP interaction mechanism identified in the JHBP-MPD complex, should provide important guidance in the search for the natural ligand of the second cavity, which is presumably important for JH delivery site recognition by JHBP.

## Materials and Methods

### Crystallization, Data Collection and Structure Determination

Mature JHBP from *Bombyx mori* was expressed as a GST-fusion protein in *Escherichia coli* and purified as described previously [14], [32]. SeMet-substituted JHBP was expressed in *E. coli* strain B834 (DE3). Crystals of JHBP in complex with MPD and SeMet derivative crystals were obtained by the hanging-drop vapor-diffusion method at 20°C from solution containing 3 µL of apoprotein at 20 mg mL^−1^ in 2 mM Tris buffer, pH 8.0, and 1.5 µL of crystallant: 25% (±)-MPD (Hampton Research, Aliso Viejo, CA, USA), 0.05 M zinc acetate (Wako Pure Chemical Industry, Osaka, Japan) and 0.1 M sodium cacodylate buffer, pH 7.0 (Hampton Research). After a week, thin plate crystal clusters appeared and a typical crystal grew to the size of dimensions 500 × 20 × 10 µm.

Diffraction data were collected at beamlines of the Photon Factory (PF), High Energy Accelerator Research Organization, Tsukuba, Japan. A single crystal was scooped up in a nylon loop and directly flash-frozen in a nitrogen stream at 95 K. Native data were collected by CCD detectors (Area Detector Systems Corp., Poway, CA, USA). Data were integrated and scaled using the program DENZO and *Scalepack* in the HKL2000 program suite [35]. MPD complex and SeMet crystals belonged to the space group *P*2_1_2_1_2_1_ and four molecules were present in the asymmetric unit with a Vm value of 3.0 Å^3^ Da^−1^ and a solvent content of 59.6% [36]. Anomalous dispersion data sets for the SeMet crystal were collected near the selenium absorption edge.

Structure solution of JHBP was performed using the SAD method. The heavy atom search and initial phase calculation were conducted using the program SOLVE/RESOLVE [37], [38]. A total of 16 heavy atom positions were determined, four of which were identified as bound zinc ions. An initial model produced by RESOLVE indicated that the crystal contains four JHBP molecules in the asymmetric unit. Successively, the structural model was refined using the MPD complex data of 2.6 Å resolution. Manual model rebuilding, introduction of water molecules, and molecular refinement were conducted using Coot [39] and Refmac5 [40]. A total of 20 zinc ions, eight MPD molecules and 121 water molecules were added into the final model. The stereochemistry of the models was analyzed with the program RAMPAGE [41]. Data collection and structure refinement statistics are summarized in Table 1. The atomic coordinates and structure factors have been deposited in the Protein Data Bank (3A1Z).

Figures of the proteins were generated by a combination of PyMOL (version 1.5: Schrödinger, LLC.) and PovRay (Version 3.7: Persistence of Vision Pty. Ltd.). Cavities on the protein surface were detected either by the Swiss-PbdViewer version 4.1 [31] or the program GHECOM [33]. The shapes of the cavities detected by GHECOM were visualized by use of OOSAWA (http://www.cfca.nao.ac.jp/~takedatk/COMPUTER/OOSAWA/oosawa.html) and PovRay.

### Competitive Ligand Binding Assay

Competitive ligand binding assays were conducted based on the method used for the JH binding assay [32] with slight modifications. For the assay, the recombinant *B. mori* JHBP (800 nM) was dissolved in 20 mM Tri-HCl buffer, pH 7.9, supplemented with 5 mM magnesium acetate, 1 mM EDTA, 1 mM DTT, 1 mM PMSF (phenylmethylsulfonyl fluoride), 2 mg mL^−1^ Leupeptides, 1 mg mL^−1^ pepstatin A, 0.1 mM diisopropyl fluorophosphate, and 10 µM 3-octylthio-1,1,1-trifluoropropanone. The protein samples were incubated for 30 min at 4°C in a volume of 100 µL containing 10 nM ^3^H-JH III (62.90 GBq mmol^−1^; New England Nuclear Chemicals) in the absence or the presence of the competitive ligand delivered in ethanol (1% v/v). Unbound JH was removed by the addition of dextran-coated charcoal solution (100 µL) and centrifugation for 2 min at 10,000 *g*. The radioactivity of a 50 µL aliquot of the supernatant was measured using Perkin-Elmer Liquid Scintillation Counter (Tri-Carb 2900TR) for quantitative determination of JHBP-bound ^3^H-JH III. Data were fitted to a hyperbolic equation describing binding to a single site [42],
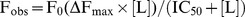
(1)where F_obs_ is the observed radioactivity at any given competitive ligand concentration, F_0_ is the radioactivity of the JHBP-bound ^3^H-JH III in the absence of competitors, and ΔF_max_ is the maximum reduction in the radioactivity. IC_50_ and ΔF_max_ are fitted as free parameters by non-linear squares regression analysis.
